# Urban Coatis (*Nasua nasua*) Exposure to *Alphainfluenzavirus influenzae*

**DOI:** 10.3201/eid3103.231640

**Published:** 2025-03

**Authors:** Bruna Hermine de Campos, Jéssica de Souza Joaquim, Nadja Simbera Hemetrio, Lara Ribeiro de Almeida, Paula Cristina Senra Lima, Grazielle Cossenzo Florentino Galinari, Marcelo Coelho Lopes, Camila Issa Amaral, Gustavo Canesso Bicalho, Beatriz Senra Santos, Nágila Rocha Aguilar, Maria Isabel Maldonado Coelho Guedes, Danielle Ferreira de Magalhães Soares, Pedro Lúcio Lithg Pereira, Cíntia Aparecida de Jesus Pereira, Walter dos Santos Lima, Camila Stefanie Fonseca de Oliveira, Roselene Ecco, Erica Azevedo Costa, Zélia Inês Portela Lobato, Marcelo Pires Nogueira de Carvalho

**Affiliations:** Universidade Federal de Minas Gerais, Belo Horizonte, Brazil (B.H. de Campos, J.S. Joaquim, L.R. de Almeida, G.C.F Galinari, M.C. Lopes, C.I. Amaral, G.C. Bicalho, B.S. Santos, N.R. Aguilar, M.I.M.C. Guedes, D.F.M. Soares, P.L.L. Pereira, C.A.J. Pereira, W.S. Lima, C.S.F. de Oliveira, R. Ecco, E.A. Costa, Z.I.P. Lobato, M.P.N. de Carvalho); Fundação de Parques Municipais e Zoobotânica de Belo Horizonte, Minas Gerais, Brazil (N.S. Hemetrio, P.C.S. Lima)

**Keywords:** influenza, viruses, Public health, immunohistochemistry, lectins, sialic acid, spillback, wildlife, urban parks, coatis, *Nasua nasua*, *Alphainfluenzavirus influenzae*, influenza A virus, Brazil

## Abstract

We detected neutralizing antibodies, viral RNA, and sialic acid receptors for *Alphainfluenzavirus influenzae* in urban coatis (*Nasua nasua*) in Brazil, suggesting exposure and susceptibility. We used hemagglutination inhibition, reverse transcription quantitative PCR, and histochemistry for detection. Increased epidemiologic wildlife surveillance would improve influenza A emergency event response.

*Alphainfluenzavirus influenzae*, also known as as influenza A virus (IAV), continues to spread globally, causing economic loss and threatening public health (*1*). IAVs can infect a range of species, leading to the emergence of new subtypes with altered host tropism or virulence (*2*). Highly pathogenic avian influenza viruses (HPAIVs) have been detected in wild animals around the world (*3*). Brazil reported its first case of HPAIV in 2023, in a *Thalasseus acuflavidus* bird (*4*).

The expression of an appropriate host cell receptor that viral haemagglutinin (HA) can bind to is the key determinant of IAV ability to infect a species (*5*). Avian influenza viruses preferentially bind to sialic acid (SA) receptors linked to galactose by α-2,3 linkage, whereas human and classical swine influenza show preference for α-2,6 linkage. Mammal hosts that co-express both SA α-2,3 and α-2,6 receptors, primarily in the upper respiratory tract, potentially play a major role in the evolution and transmission of IAVs. Susceptibility to infection by IAVs of different origins (human, avian, or swine) can support rearrangement between IAVs and contribute to the emergence of genetically diverse viruses (*6*).

Coatis (*Nasua nasua*) are carnivores of the Procyonidae family (*7*). Coatis are susceptible to different virus infections, such as SARS-COV-2, and can be sentinels for animal, human, and environmental health (*8*). We investigated coatis IAV exposure and susceptibility from an urban park (Appendix Figure 1), which comprises an intersecting area of urban and wild environments, in Belo Horizonte, Brazil.

During 2013, 2014, 2018, 2019, and 2021, we collected samples from wild coatis. We captured coatis respecting Biosafety standards and using personal protective equipment. Ethical approvals were obtained for research development (Appendix). We placed tomahawk (Zootech, https://zootechonline.com.br/armadilhas) traps at strategic points and checked them daily. We physically examined the captured coatis and then gave each an intramuscular injection of Zoletil 100 (Virbac, https://us.virbac.com) at a dose of 7–10 mg/kg. We collected blood samples from the coatis and identified each with a subcutaneous microchip before releasing them.

We collected whole blood samples at a limit of 1% of bodyweight by jugular venipuncture from 145 coatis (Appendix Table). For 63 coatis captured in 2021, we also collected oropharyngeal swab samples and packed them in 3 mL of buffered saline solution with penicillin (200 U/mL) and streptomycin (200 μg/mL). We stored serum samples at −20°C and swabs at −80°C. We used dead coatis (n = 3) found in the park for tissue sample collection. We fixed tissues in 10% buffered formalin, embedded them in paraffin, and sectioned the tissue samples at 4 μm thickness.

We conducted hemagglutination inhibition (HI) assays to detect neutralizing antibodies to IAV (Appendix). We identified antibodies in 92.4% (n = 134) of the samples. Influenza A(H1N1)pdm09 subtype was detected in coatis’ samples from each year of the study period. H3N2 virus was detected in samples from 2018, 2019, and 2021, and seasonal human H1N1 virus was detected in 2021 (Appendix Figure 2). None of the captured coatis demonstrated any clinical manifestations of illness.

We performed RNA extraction from swabs by using QIAamp MinElute Virus Spin Kit (QIAGEN, https://www.qiagen.com), and quantitative reverse transcription PCR for universal and subtype detection of IAVs (Appendix). We detected viral RNA in 15.87% of samples from 2021 (Table). We detected subtype H3N2 genetic material from coati 347.

**Table:** Urban coatis (*Nasua nasua*) samples positive for *Alphainfluenzavirus influenzae* viral RNA, demonstrating exposure and susceptibility, Brazil

Coati identification no.	Collection date	Sex	Cycle threshold
343	2021 Jan 18	F	36.7
344	2021 Jan 18	M	37.3
518	2021 Jan 18	M	38.3
520	2021 Jan 18	M	36.6
347	2021 Jun 28	F	37.776
541	2021 Jun 30	F	34.266
531	2021 Jul 9	M	37.436
354	2021 Aug 13	M	38.880
355	2021 Aug 13	F	38.824
356	2021 Aug 13	M	35.462

To detect α-2,6 and α-2,3 SA receptors, we selected nasal conchae, trachea, and lung tissue for lectin histochemistry technique by using *Maackia amurensis* and *Sambucus nigra* plant lectin (Appendix). We detected positive labels for those receptors in all analyzed tissues. The receptor marking was visualized as a strong brown color at the apical membrane of the nasal ephitelium and ciliated cells of the respiratory tract (nasal turbinate, trachea, bronchus and bronchiole), including globet cells, pneumocytes, and pulmonary endothelial cells (Figure). The 2 lectins labeled both receptors with diffuse distribution in the respiratory tissues.

**Figure:**
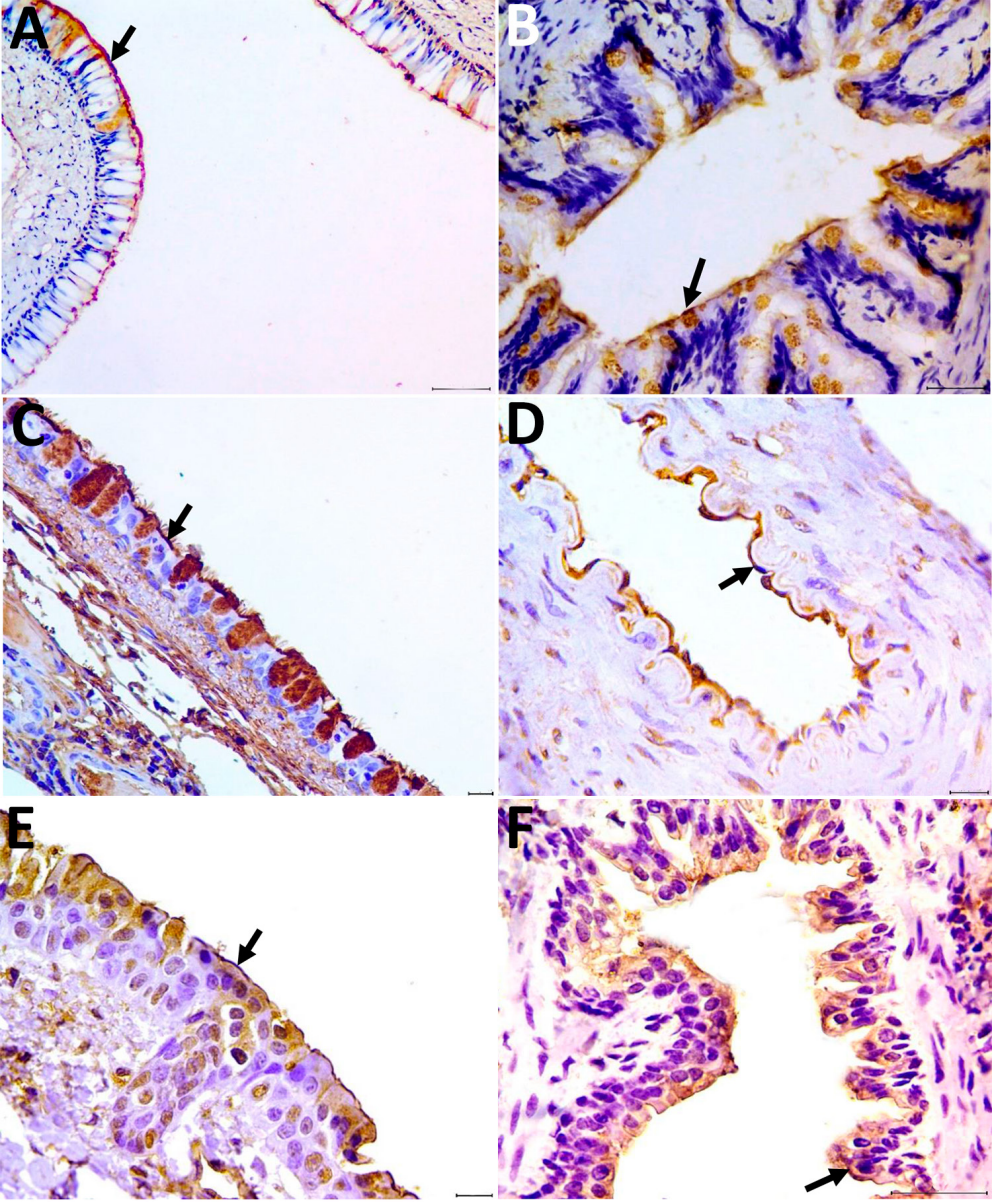
Detection of α-2,3 and α-2,6 receptors in tissues from the respiratory system of coatis (*Nasua nasua*), Brazil. A–C) Arrows indicate labeling of the α-2,3 receptor in the ciliated epithelium for the lectin *Maackia amurensis* II of the nasal concha (A), lung (bronchiole) tissue (B), and trachea (C). Scale bars = 100 µm in panel A, 50 µm in panel B, and 20 µm in panel C. D–F) Arrows indicate labeling of the α-2,6 receptor in the endothelium for *Sambucus nigra* lectin in the arteriole (D), rostral concha (E), and lung (bronchiole) (F). Scale bars = 20 µm in panels D and E, 50 µm in panel F. Tissue was counterstained with hematoxylin and revealed with diaminobenzidine chromogen.

The detection of antibodies against IAV subtypes suggests natural exposure of coatis to IAVs. We were unable to confirm the mode of IAV transmission to coatis; nevertheless, we found evidence of close contact of coatis to contaminated human waste and food, indicating the possibility of human-to-animal transmission.

The seasonal human H1N1 virus subtype, which circulated in Brazil during 2001–2003, was detected in swab samples, suggesting the possible dissemination, maintenance, and transmission capacity of coatis. Those results agree with previously published reports that detected the same viral subtype in wild carnivores during 2009–2011 (*9*). In 2021 and 2022, there were reports of outbreaks in Brazil triggered by the emergence of a new influenza A(H3N2) strain, named Darwin, occurring concurrently with SARS-CoV-2 as co-infection (*10*). The presence of α-2,6 and α-2,3 SA receptors highlight the possibility of co-infection of coatis with different viral lineages, giving the animals a potential role in IAV spillover events. Because of urban coatis’ habitats, the absence of signs of clinical illness, and the recent introduction of HPAIV into Brazil, a heightened epidemiologic wildlife surveillance strategy would improve the ability to respond to IAV emergency health events.

Appendix Additional information about urban coatis (*Nasua nasua*) exposure to *Alphainfluenzavirus influenzae*.
