# Supplementary material for: Urban Coatis (Nasua nasua) Exposure to Alphainfluenzavirus influenzae
Source: Emerg Infect Dis. 2025 Mar;31(3):621–4. doi: 10.3201/eid3103.231640 (PMC11878311; doi:10.3201/eid3103.231640)
Supplement: Appendix — Additional information about urban coatis (Nasua nasua) exposure to Alphainfluenzavirus influenzae. [file 23-1640-Techapp-s1.pdf]

Article DOI: https://doi.org/10.3201/eid3103.231640

EID cannot ensure accessibility for supplementary materials supplied by authors. Readers who have difficulty accessing supplementary content should contact the authors for assistance.

Urban Coatis (*Nasua nasua*) Exposure to *Alphainfluenzavirus influenzae*

Appendix

Ethics Approval

The study was approved by SISBIO (Sistema de Autorização e informação em Biodiversidade, license numbers 39817–1, 61968–1 and 75831), Ethical Committee on Animal Use (CEUA) of Universidade Federal de Minas Gerais (protocol numbers 274/2013, 306/2017, 158/2020 and 100/2021), SisGen (Sistema Nacional de Gestão do Patrimônio Genético, no. A627307), and Fundação de Parques Municipais e Zoobotânica de Belo Horizonte (protocol number FU 004–2020).

Methods

The Hemagglutination Inhibition (HI) assay, a well-established laboratory method for the classification and subtyping of hemagglutinating viruses, was conducted. Serum samples were heat inactivated at 56°C for 30 min, treated with a 20% kaolin suspension (Sigma-Aldrich, St. Louis, MO), and absorbed with 0.5% turkey red blood cells (RBCs) (*1*). The H1N1pdm09, GenBank accession number JQ666849; human seasonal H1N1hu, GISAID accession number EPI_ISL_575027 and H3N2, GISAID accession number EPI_ISL_574871 were used as antigens in the HI assays. In 96-well V-bottom microtitration plates, 25 µl of 1X PBS were added to all wells. In the first row (A), 25 µl of treated serum were added to each well (1:20 dilution), and serial dilutions were performed throughout the plate in base 2, up to a final dilution of 1:2560. To all wells, except for the serum control, 25 µl of viral suspension with 4 hemagglutinating units of the virus (4 HAU) were added. The plates were incubated at room temperature for 30 minutes.

After this period, 25 µl of 0.8% turkey red blood cell suspension in 1X PBS were added to all wells, and incubation was carried out at room temperature for 30 minutes. At the end of

this period, the test was read. Viral back-titration was performed to confirm the amount of virus used in the Hemagglutination technique, which should be 4 HAU.

The antibody titers of each sample were determined as the inverse of the highest dilution in which hemagglutination was not observed. Titers equal to or less than 20 were considered negative, between 40 and 80 were considered low titers, between 160 and 320 were considered medium titers, and titers greater than or equal to 640 were considered high (1).

For screening and subtyping the Influenza A virus, protocols and a set of primers described by WHO for human influenza surveillance (London WHO CC; January 2020) were used. RT-qPCR assays were done to detect Influenza type A viruses (Matrix gene) (Universal IAV Screening), A(H1N1) pdm09 viruses (HA gene), Former seasonal influenza A(H1N1) (HA gene), A(H3N2) viruses (HA gene), and A(H5N1) viruses (HA gene) (*2*). To determine the limit of detection (LOD) for the Universal IAV Screening RT-qPCR assay, tenfold dilutions of Influenza A (H1N1) pdm09 isolate, containing $1x10^7$ PFU/mL, were also analyzed. The repeatability (intra-assay variance) and reproducibility (inter-assay variance) of the assays were assessed using tenfold dilutions of the Influenza A isolates (H1pdm, H1hu and H3) and for NA (N1pdm, N1hu and N2). The assays were performed in triplicate and in three different runs to evaluate the coefficients of variation (CVs) of both assays. Intra- and inter-assay CVs based on the Cq values were calculated using Statistical Analysis System software. Each RT-qPCR 96-well plate had 3 positive controls, 3 negative controls, 3 positive extraction controls, and 3 negative extraction controls. We ran the RT-qPCR on a QuantStudio 5 Real-Time PCR System and analyzed data using QuantStudio 5 Real-Time PCR System Software (Thermo Fisher Scientific). Consistent with LOD, we considered samples with cycle threshold (Ct) values <39.0 to be positive for the virus, if the repeat results were the same as before. The specimen was considered negative if the Ct value was undetectable.

Three dead coatis found in the MMP area in 2021 were collected and transported at 4°C for postmortem examination and collection of tissue samples. Representative tissue samples were fixed in 10% buffered formalin for 52 h and histologic procedures were performed. For the lectin histochemistry technique, sections of nasal conchae, trachea and lungs were used to detect α-2,6 and α-2,3 SA receptors in the three coatis. We used specific lectins (*Maackia amurensis* and *Sambucus nigra*) as standardized in previous studies (*3–5*). Swine trachea and chicken lungs

were used as positive controls for the detection of α-2.6 and α-2.3 receptors, respectively. The positive controls underwent identical histological preparation and histochemistry procedures for lectins as the coati samples. In the negative controls, the slides were incubated with PBS instead of lectins.

References

1. World Health Organization. WHO. (2011) WHO Global Influenza Surveillance Network Manual for the laboratory diagnosis and virological surveillance of influenza. 2nd ed. Geneva: World Health Organization.

2. World Health Organization. WHO information for the molecular detection of influenza viruses. 2021. [cited 2022 Feb 18]. https://cdn.who.int/media/docs/default-source/influenza/molecular-detention-of-influenza-viruses/protocols_influenza_virus_detection_feb_2021.pdf

3. Costa T, Chaves AJ, Valle R, Darji A, van Riel D, Kuiken T, et al. Distribution patterns of influenza virus receptors and viral attachment patterns in the respiratory and intestinal tracts of seven avian species. Vet Res. 2012;43:28. PubMed https://doi.org/10.1186/1297-9716-43-28

4. Hall JS, Bentler KT, Landolt G, Elmore SA, Minnis RB, Campbell TA, et al. Influenza infection in wild raccoons. Emerg Infect Dis. 2008;14:1842–8. PubMed https://doi.org/10.3201/eid1412.071371

5. Ning ZY, Wu XT, Cheng YF, Qi WB, An YF, Wang H, et al. Tissue distribution of sialic acid-linked influenza virus receptors in beagle dogs. J Vet Sci. 2012;13:219–22. PubMed https://doi.org/10.4142/jvs.2012.13.3.219

Appendix Table. Numbers of captured coatis categorized by collection date, sex, and age*

	Male					Female					
Year	Infant	Young	Immature adult	Adult	Total	Infant	Young	Immature adult	Adult	Total	Total
2013	1	1	0	4	6	1	6	0	13	20	26
2014	7	0	0	2	9	4	1	0	5	10	19
2018	0	7	6	2	15	0	3	4	3	10	25
2019	0	2	3	0	5	0	5	0	2	7	12
2021	9	4	4	8	25	4	4	8	22	38	63
Total	17	14	13	16	60	9	19	12	45	85	145

*Collection comprised 145 coatis (*Nasua nasua*) (58.62% female and 41.38% male) and 14 additional recaptures. Live traps (n = 25) were placed at 5 strategic points of passage for coatis (5 traps set per point) in Mangabeiras Municipal Park in Belo Horizonte, Brazil. The 5 capture points were: Caraça (n = 69), Casa de bonecas (n = 23), Mirante (n = 5), Praça das águas (n = 29), and trilha 12 (n = 19). Biometrics analyses were performed to estimate age.

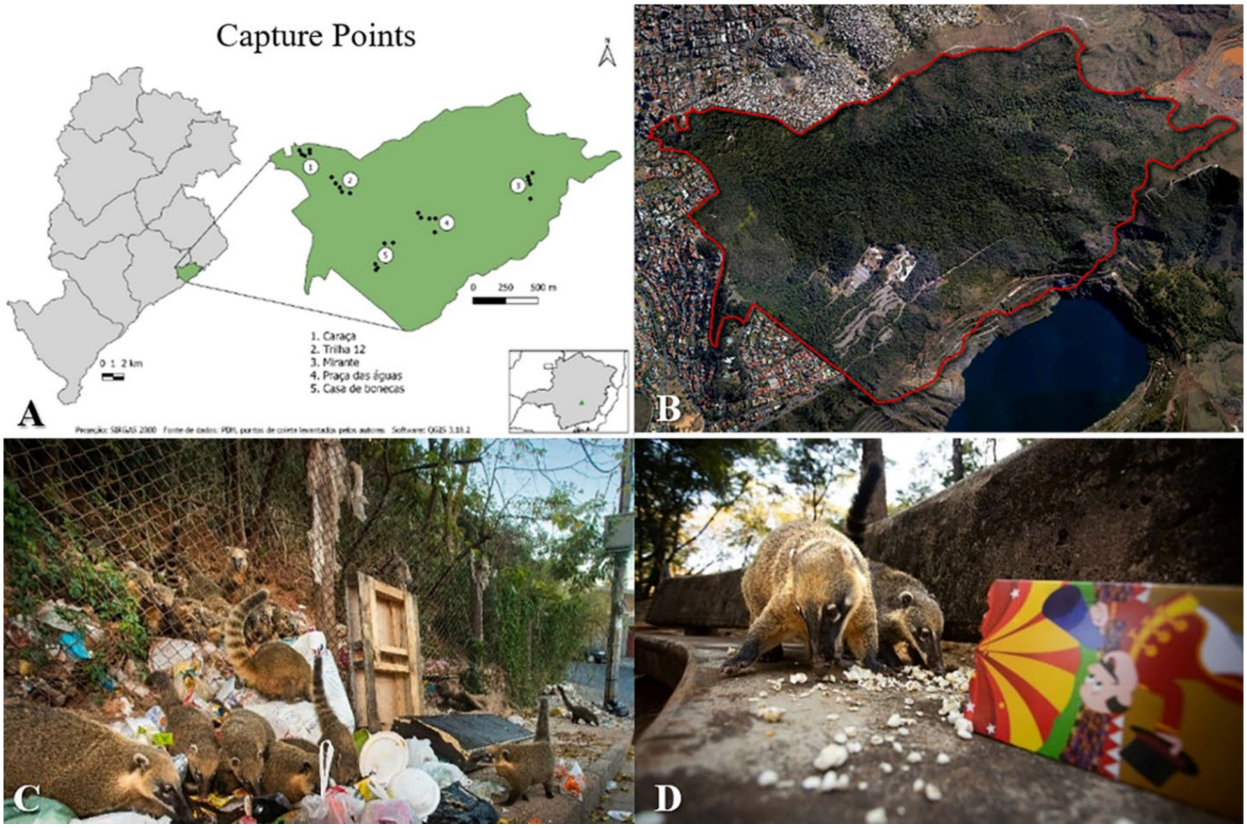

Appendix Figure 1. Panorama of Mangabeiras Park in southeastern Brazil in a study of Influenza A viruses in the coati population. A) Map of Belo Horizonte highlighted in Minas Gerais, emphasizing Mangabeiras Municipal Park (19°56′ S and 43°54′ W), indicating the five locations where coatis were captured. B) Satellite image of Mangabeiras Municipal Park (encircled by a red line) in the city of Belo Horizonte, highlighting the forested area in contrast to the urbanized area, and the presence of ore extraction. C and D) Coatis in Mangabeiras Park in contact with human waste and food remnants. Photographer: Augusto Gomes.

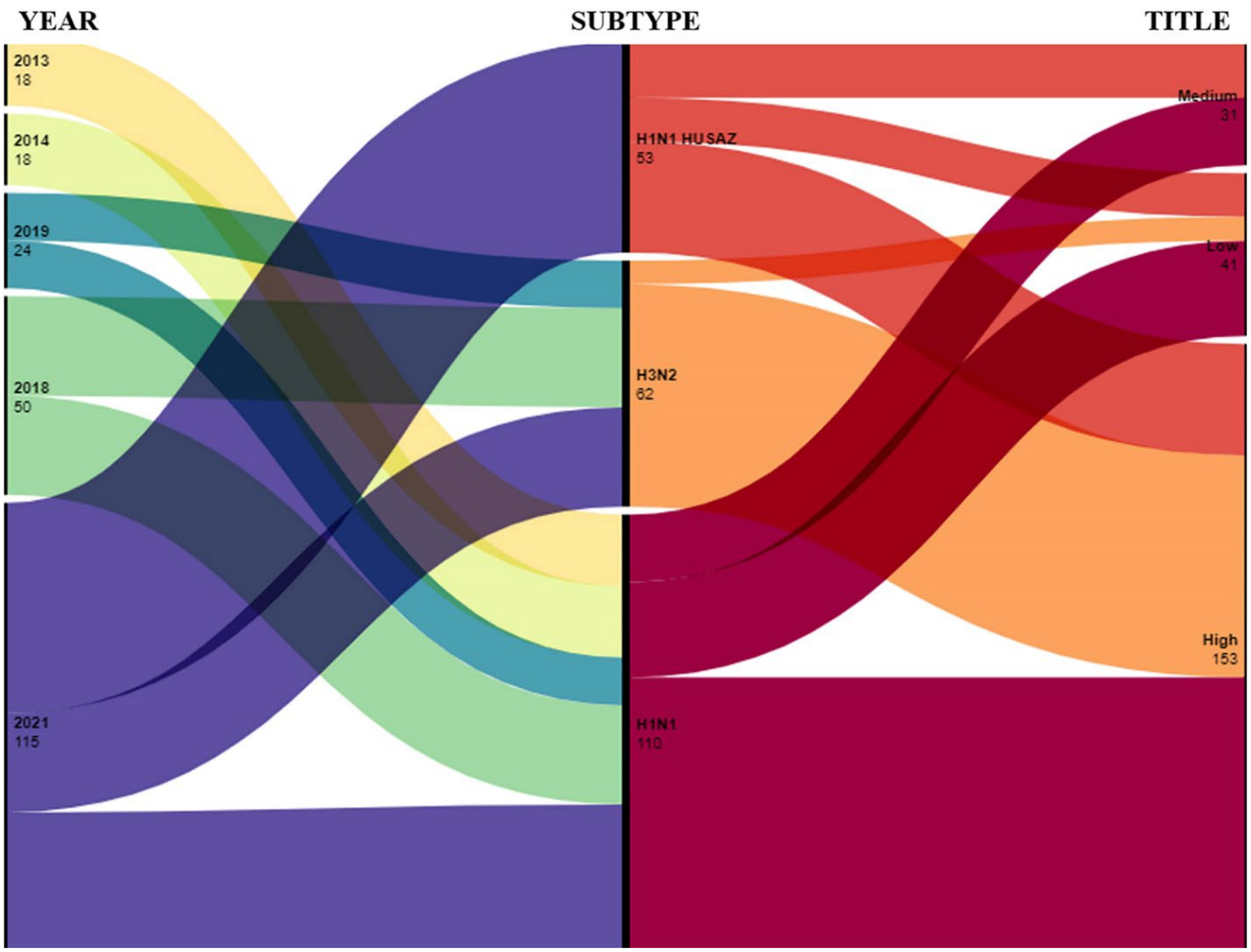

Appendix Figure 2. Chart of the distribution of seropositivity scores for Influenza A subtypes H1N1pdm09, seasonal H1N1hu, and H3N2 among coatis (*Nasua nasua*). Serum samples from coatis (n = 145) from the years 2013, 2014, 2018, 2019, and 2021 were analyzed through Hemagglutination Inhibition. Titers equal to or less than 20 were considered negative, titers between 40 and 80 as low, titers between 160 and 320 as medium, and titers greater than or equal to 640 as high. Among the animals that tested seropositive for H1N1pdm09 (n = 110), 62.7% exhibited high titers, 15.5% exhibited medium titers, and 21.8% exhibited low titers. Among the animals seropositive for seasonal H1N1hu (n = 53), 52.8% exhibited high titers, 26.4% exhibited medium titers, and 20.8% exhibited low titers. Among those coatis that tested seropositive for H3N2 (n = 62), 90.3% exhibited high titers, and 9.7% exhibited low titers. Neutralizing antibodies to Influenza A viruses were detected in 134 samples (134/145; 92.4%), with the H1N1pdm09 subtype present throughout the sampled periods (69/134; 51.5%), H3N2 occurring in 2018, 2019, and 2021 (56/134; 41.8%), and seasonal human H1N1 in 2021 (28/54; 51.9%).
